# 

**Published:** 1875-01

**Authors:** 


Vol. 22. JANUARY, 1875. No. 1.

TWENTY-SECOND YEAR.

52

### HALL'S
### Journal of Health.

W. W. HALL, M. D., EDITOR.

PUBLISHED BY THE AMERICAN AND FOREIGN PUBLICATION CO., 137 EIGHTH ST.. NEW YORK.

Agents for the Trade :

AMERICAN NEWS COMPANY, 121 NASSAU STREET.
NEW YORK NEWS COMPANY, 18 BEEKMAN STREET.

Terms, Two Dollars a Year.

SINGLE NUMBER, 20 CENTS.

